# Identifying vertebral fractures in the Japanese population using the trabecular bone score: a cross-sectional study

**DOI:** 10.1186/s12891-022-05839-z

**Published:** 2022-11-11

**Authors:** Yasuyuki Omichi, Noriaki Mima, Ryo Okada, Keizo Wada, Masatoshi Morimoto, Koichi Sairyo

**Affiliations:** 1 Department of Orthopedics, Tokushima University, 3-18-15 Kuramoto, 770-8503 Tokushima, Japan; 2 Department of Orthopedics, Mima Hospital, Tokushima, Japan; 3 Department of Orthopedics, Tokushima Prefectural Miyoshi Hospital, Tokushima, Japan

**Keywords:** Trabecular bone score, Vertebral fracture, Osteoporosis, Japanese population

## Abstract

**Background:**

The trabecular bone score (TBS) is reported to be an independent predictor of fracture risk in patients with primary or secondary osteoporosis. However, there have been few reports on its use in the Japanese population. This study aimed to investigate the risk factors for vertebral fracture in the Japanese population and to evaluate the usefulness of TBS.

**Methods:**

This cross-sectional study involved 279 patients aged 60–90 years in whom bone mineral density (BMD) was measured by dual-energy X-ray absorptiometry (DXA). TBS was calculated based on the DXA scans. The presence or absence of vertebral fractures was assessed from T11 to L5. The patients were divided into those with vertebral fractures (VF group, n = 104) and those without vertebral fractures (non-VF group, n = 175).

**Results:**

Of the 104 patients in the VF group, 75 had 1 vertebral fracture and 29 had 2 or more fractures. The mean TBS was 1.28 in the VF group and 1.35 in the non-VF group (*p* < 0.001). The mean BMD values at the lumbar spine and femoral neck were lower in the VF group (*p* < 0.001). The areas under the receiver-operating characteristic curve for incidence of vertebral fractures were 0.700, 0.737, and 0.689 for TBS, lumbar spine BMD, and femoral neck BMD, respectively. Multiple logistic regression analysis identified lumbar spine BMD, TBS, and female sex as significant risk factors for vertebral fractures. The proportion of patients in the group with osteoporosis or osteopenia who had vertebral fractures was higher in those with a low TBS (≤ 1.23) than in those with a non-low TBS (> 1.23).

**Conclusion:**

TBS was a significant indicator of vertebral fractures in the Japanese population and might contribute to identifying patients with vertebral fractures, particularly those with osteopenia who need pharmacologic therapy.

**Supplementary Information:**

The online version contains supplementary material available at 10.1186/s12891-022-05839-z.

## Background

Osteoporosis is a systemic skeletal disease characterized by low bone mass and microarchitectural deterioration of bone, leading to bone fragility and increased risk of fracture [1, 2]. According to the World Health Organization (WHO) criteria, osteoporosis is defined as a bone mineral density (BMD) that is ≤ 2.5 standard deviations (SD) below the average value for healthy young adults (T-score − 2.5 SD or less) [3, 4]. The most widely validated technique for measuring BMD is dual-energy X-ray absorptiometry (DXA), accurately estimates BMD [5–8].

However, DXA has some shortcomings. For example, it does not detect textural deterioration of bone tissue, and vertebral fractures and degenerative spondylolysis falsely increase lumbar BMD readings on DXA [4, 9].

Trabecular bone score (TBS) is a new gray-level bone textural index of trabecular bone structure derived from the anteroposterior DXA image of the lumbar spine [10, 11]. TBS is related to bone microarchitecture and provides skeletal information that is not captured by standard BMD measurements [12]. In patients with secondary osteoporosis, including those with diabetes mellitus, chronic renal failure, cirrhosis, or ankylosing spondylitis and those on steroids, TBS could provide more information on microarchitecture than BMD [13–20]. Even in patients with primary osteoporosis, TBS is reported to be an independent predictor of fractures [12, 21, 22], with lower TBS values associated with a higher fracture risk [23–25]. Furthermore, TBS has been shown to improve the accuracy of prediction for major osteoporotic fractures and hip fractures when using the Fracture Risk Assessment Tool (FRAX) [26, 27], and is now used with the FRAX in clinical practice [28].

Reports on TBS have increased in recent years but some points remain unclear. Although the average TBS varies according to ethnicity, there have been few reports on the TBS in the Japanese population with vertebral fractures. Moreover, there is almost no mention of TBS in the latest (2015) Japanese treatment guideline for osteoporosis. Therefore, this study aimed to investigate the risk factors for vertebral fractures in the Japanese population and to evaluate the usefulness of TBS.

## Methods

### Patients

**Fig. 1:**
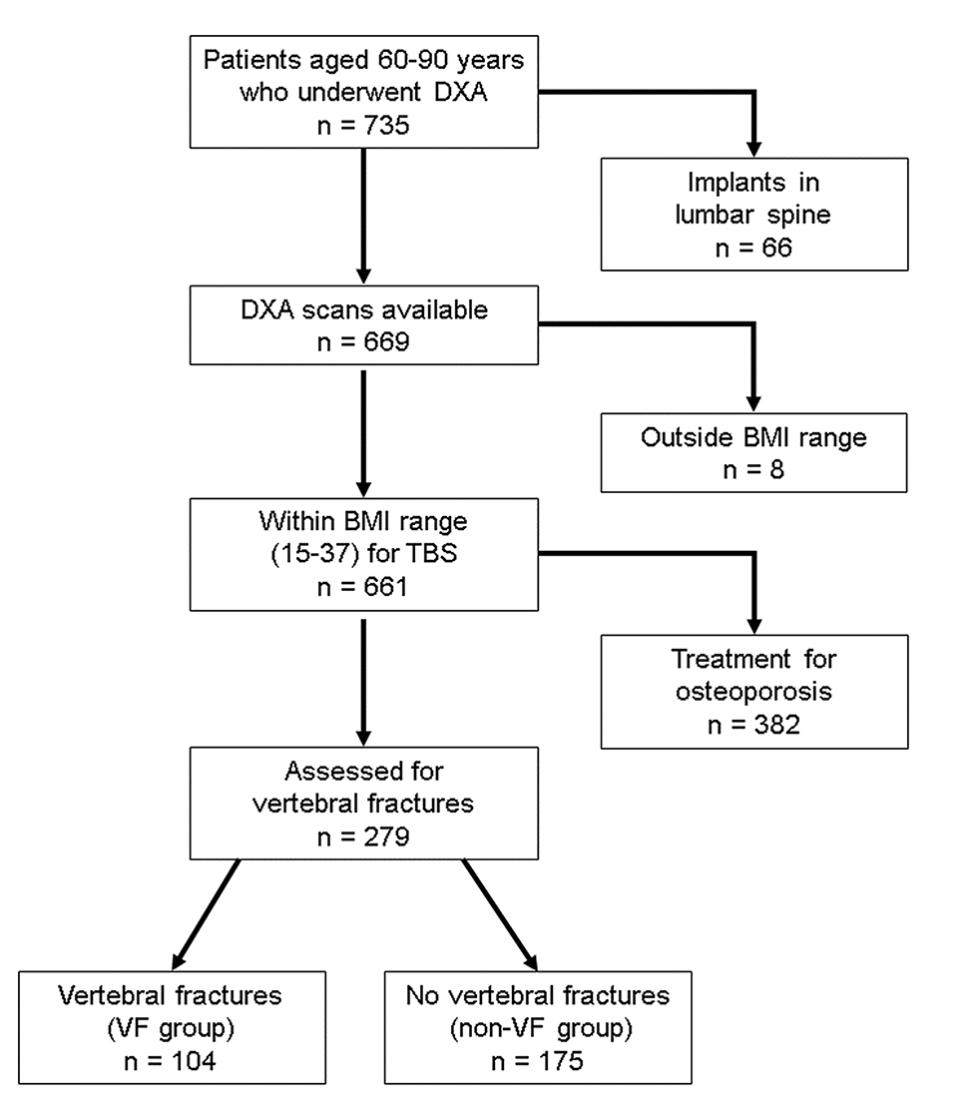
Flow chart showing the study enrollment process. BMI, body mass index; DXA, dual-energy X-ray absorptiometry; TBS, trabecular bone score

This cross-sectional study involved patients aged 60–90 years who underwent DXA at Mima Hospital between August 2019 and April 2021. Of 735 patients considered for inclusion in the study, 66 were excluded because of implants in the lumbar spine, affecting DXA’s accuracy. According to the manufacturer of the TBS software, TBS is accurate for patients with a body mass index (BMI) in the range of 15–37 kg/m^2^. Eight individuals with a BMI outside the range were also excluded. To rule out the effects of osteoporosis drugs, a further 382 patients who were taking a bisphosphonate, denosumab, teriparatide, romosozumab, a selective estrogen receptor modulator, vitamin D, vitamin K, or calcium were excluded. Finally, 279 patients were included in the analysis. The patients were divided into those with vertebral fractures (VF group) and those without vertebral fractures (non-VF group) (Fig. 1). Vertebral fractures were diagnosed based on radiographic findings. Lateral X-ray images of the thoracolumbar spine were obtained in the standing position at the same clinic visit when the DXA scans were acquired. The extent of the radiographic evaluation was from T11 to L5. The presence or absence of vertebral fractures was assessed in this range. We graded the severity of fractures using Genant’s semiquantitative (SQ) method (loss of height of the anterior, middle, posterior, or whole vertebrae) [29]. For the purposes of this study, we defined vertebral fracture as SQ1–SQ3 (≥ 20% height loss). Two orthopedic specialists (Dr. Omichi, Dr. Mima) diagnosed the vertebral fractures while working independently on separate days. We calculated the intraclass correlation coefficients for intraobserver and interobserver reliability, both of which were high (0.88 and 0.89, respectively). Information on age, sex, height, weight, BMI, past medical history (including type 2 diabetes mellitus, rheumatoid arthritis, and treatment with steroids) was obtained retrospectively from the medical records. The study was approved by the ethics committee of Mima Hospital (approval No. R2021.6-1). Informed consent was obtained from all patients.

### Measurement of BMD

BMD was measured at the lumbar spine (L2–L4) and femoral neck using a DXA system (Horizon; Hologic, Inc., Marlborough, MA). Fractured vertebra or vertebra with a T-score > 1 with respect to the previous or successive vertebra were excluded from analysis, according to the exclusion criteria recommended by the International Society for Clinical Densitometry (ISCD) (30). In accordance with the WHO criteria, osteopenia was defined as a BMD T-score between − 1 and − 2.5 and osteoporosis as a T-score of − 2.5 or less [31].

### Measurement of TBS

TBS was measured using optional DXA software (TBS iNsight version 3.1; Medimaps Group SA, Geneva, Switzerland) and scored on the same anteroposterior DXA scan of the spine used to measure BMD. TBS was calculated as the average score for the vertebral bodies at L2–L4. Vertebrae mentioned in the section on measurement of BMD was excluded from the analysis. In accordance with a meta-analysis of fracture risk assessment as a function of TBS that included 14 prospective cohorts [32], the patients were divided into a low TBS group (TBS ≤ 1.23), an intermediate TBS group (TBS 1.23–1.31), and a high TBS group (TBS ≥ 1.31).

### Statistical analysis

All data are expressed as the mean ± standard deviation. Characteristics of patients with or without vertebral fractures were compared using the Student’s *t*-test or Mann–Whitney *U* test or Fisher’s exact test as appropriate. The accuracy of TBS and BMD for discrimination of vertebral fractures was assessed by determining areas under the receiver-operating characteristic curve (AUCs). All statistical analyses were performed using SPSS version 27 software (IBM Corp., Armonk, NY). A *p*-value < 0.05 was considered statistically significant.

## Results

Of the 104 patients in the VF group, 75 had 1 vertebral fracture, 18 had 2 fractures, and 11 had 3 or more fractures. Vertebral fractures were most common at the T12–L2 level and thoracolumbar junction, followed by the lower lumbar spine.

Table 1 shows comparisons of anthropometric and demographic characteristics between the VF group and non-VF group. The VF group was older, shorter, and lighter than the non-VF group (*p* < 0.05). There was no significant difference between the two groups in the proportion of women (*p* = 0.59) or in the proportions with diabetes mellitus, rheumatoid arthritis, and a history of steroid use (*p* = 0.85, *p* = 0.57, and *p* = 0.46, respectively).

**Table 1:** Comparison of patient characteristics according to vertebral fracture status

	VF group	Non-VF group	***p***-value
Number	104	175	
Age (years) (mean ± SD)	79.9 ± 7.7	76.0 ± 8.2	< 0.001
Female sex (*n*, %)	75 (72.1%)	120 (68.6%)	0.59
Height (cm) (mean ± SD)	151.4 ± 10.1	153.9 ± 7.8	0.03
Weight (kg) (mean ± SD)	50.9 ± 11.2	55.8 ± 10.3	< 0.001
BMI (kg/m^2^) (mean ± SD)	22.0 ± 3.4	23.5 ± 3.8	0.003
Type 2 DM (*n*, %)	12 (11.5%)	23 (13.1%)	0.85
Rheumatoid arthritis (*n*, %)	14 (13.5%)	19 (10.9%)	0.57
Steroid use (*n*, %)	9 (8.7%)	10 (5.7%)	0.46
TBS (L2–4) (mean ± SD)	1.28 ± 0.11	1.35 ± 0.09	< 0.001
High (≥ 1.31) (*n*, %)	36 (32.4)	112 (64)	< 0.001
Intermediate (1.23–1.31) (*n*, %)	34 (32.4)	53 (30.3)	0.69
Low (≤ 1.23) (*n*, %)	34 (34.3)	10 (5.7)	< 0.001
BMD (lumbar spine) (g/cm^2^) (mean ± SD)	0.76 ± 0.17	0.92 ± 0.20	< 0.001
T-score (lumbar spine) (mean ± SD)	−2 ± 1.4	−0.6 ± 1.8	< 0.001
Normal (*n*, %)	20 (19.2)	96 (54.9)	< 0.001
Osteopenia (*n*, %)	39 (37.1)	54 (30.9)	0.29
Osteoporosis (*n*, %)	45 (42.9)	25 (14.3)	< 0.001
BMD (femoral neck) (g/cm^2^) (mean ± SD)	0.51 ± 0.13	0.60 ± 0.13	< 0.001
T-score (femoral neck) (mean ± SD)	−3.2 ± 1.4	−2.2 ± 1.5	< 0.001
Normal (*n*, %)	5 (5.1)	28 (16.1)	< 0.001
Osteopenia (*n*, %)	22 (22.4)	64 (36.8)	0.02
Osteoporosis (*n*, %)	71 (72.5)	82 (47.1)	< 0.001

BMD, bone mineral density; BMI, body mass index; DM, diabetes mellitus; SD, standard deviation; TBS, trabecular bone score

**Fig. 2:**
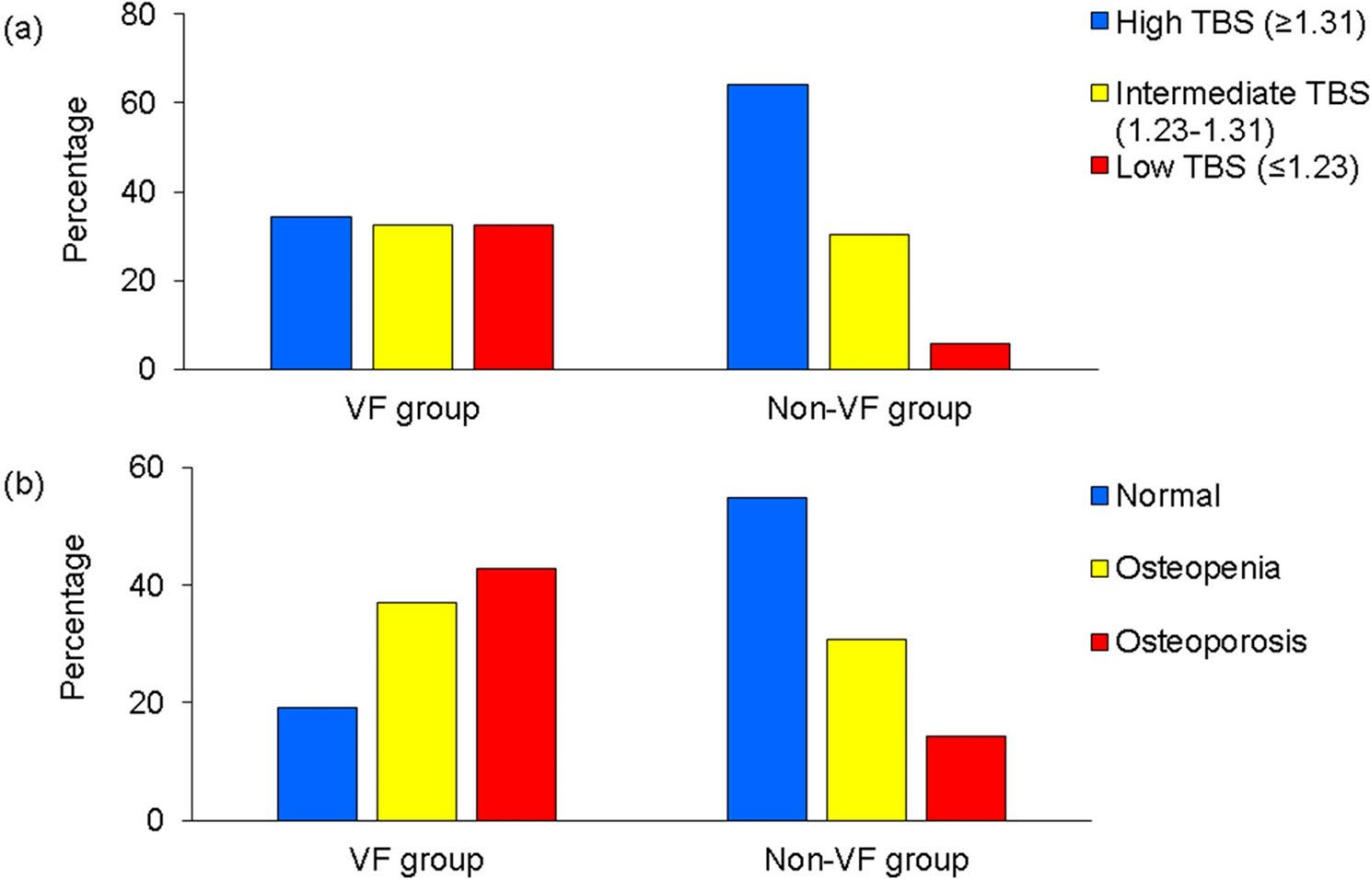
(**a**) Percentages according to TBS in the VF group and the non-VF group. The proportion of patients with low TBS was higher in the VF group than in the non-VF group (*p* < 0.001). (**b**) Percentages according to the BMD T-score at the lumbar spine in the VF group and the non-VF group. The proportion of patients with osteoporosis was higher in the VF group than in the non-VF group (*p* < 0.001). BMD, bone mineral density; TBS, trabecular bone score; VF, vertebral fracture

**Fig. 3:**
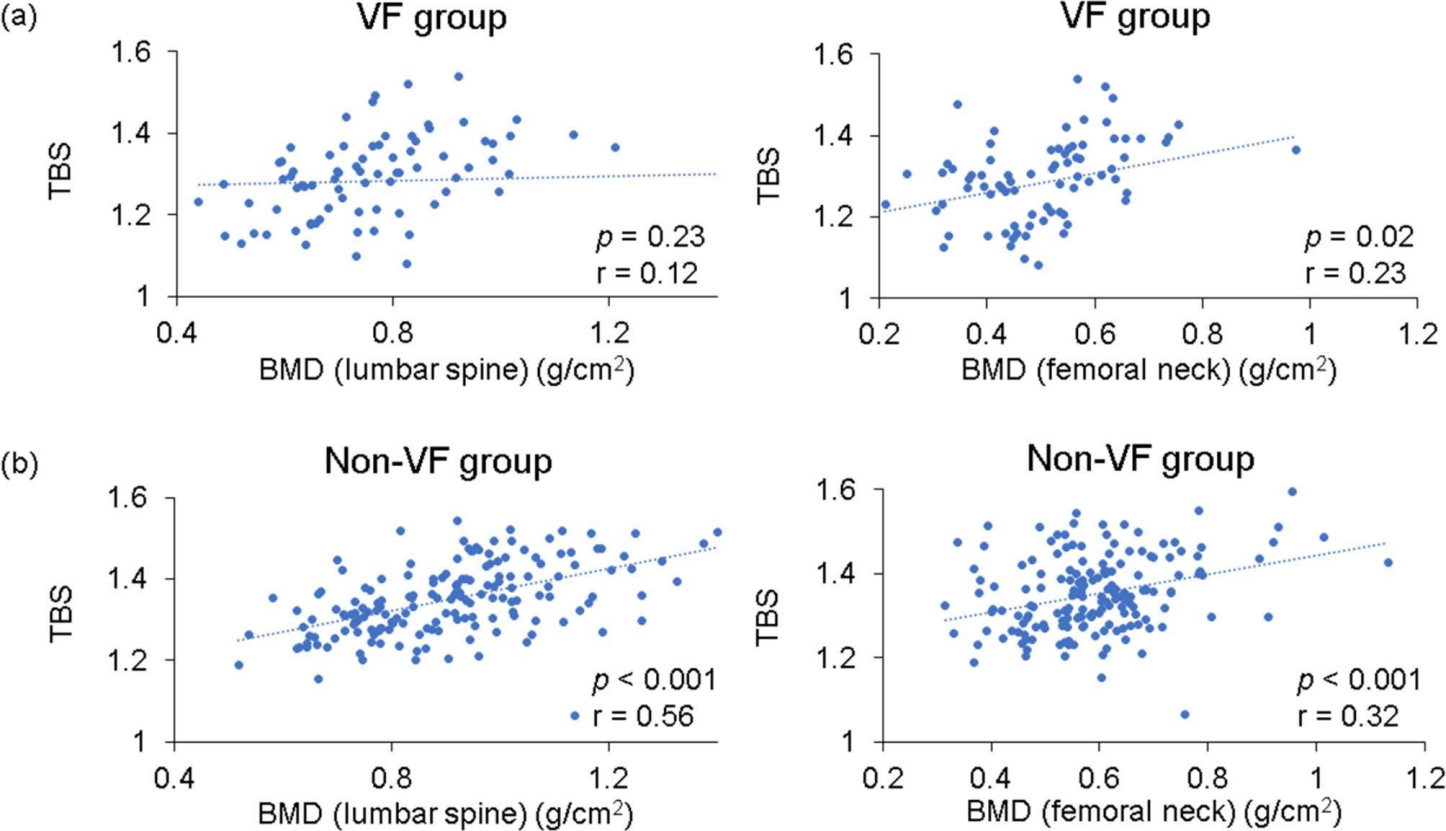
Correlation between BMD and TBS in the (**a**) VF group and (**b**) non-VF group. BMD was positively correlated with TBS (*p* < 0.01). BMD, bone mineral density; TBS, trabecular bone score; VF, vertebral fractures

Mean TBS was 1.28 in the VF group and 1.35 in the non-VF group (*p* < 0.001). The proportion with la ow TBS (≤ 1.23) was higher in the VF group (*p* < 0.001; Fig. 2a). Mean BMD at the lumbar spine was 0.76 g/cm^2^ in the VF group and 0.92 g/cm^2^ in the non-VF group (*p* < 0.001); mean BMD at the femoral neck was 0.51 g/cm^2^ and 0.60 g/cm^2^, respectively (*p* < 0.001) (7 with implants in bilateral hips were excluded). The proportion of patients with osteoporosis (lumbar spine T-score − 2.5 or less) was higher in the VF group than in the non-VF group (*p* < 0.001; Fig. 2b). There was no correlation between lumbar spine BMD and TBS in the VF group (*r* = 0.12, *p* = 0.23); however, there was a strong correlation in the non-VF group (*r* = 0.56, *p* < 0.001) (Fig. 3). BMD at the femoral neck was weakly correlated with the TBS (VF group: *r* = 0.23, *p* = 0.02; non-VF group: *r* = 0.32, *p* < 0.001).

**Fig. 4:**
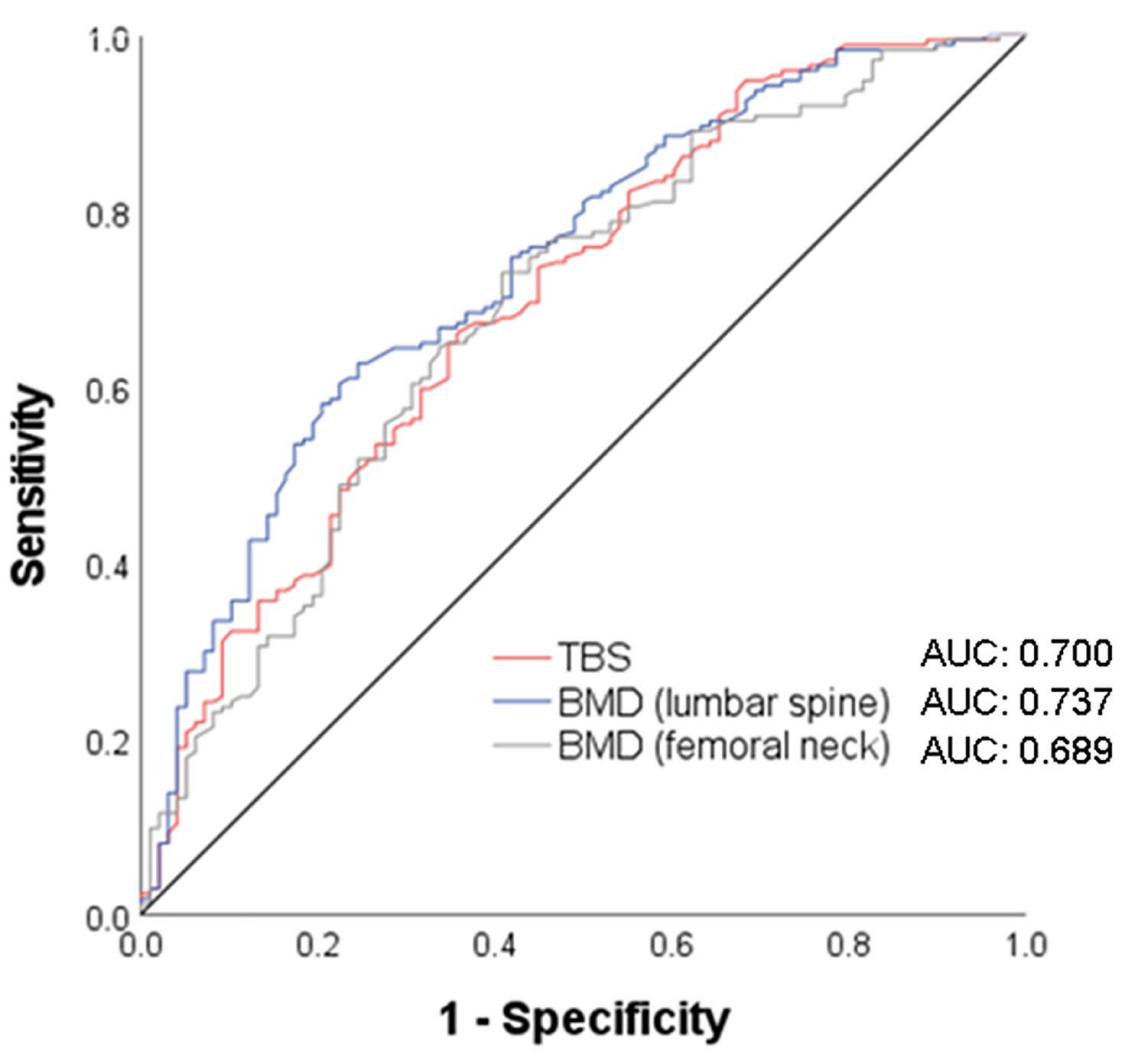
Receiver-operating characteristic curves for incidence of vertebral fractures according to BMD and TBS in the entire study population. AUC, area under the curve; BMD, bone mineral density; TBS, trabecular bone score

AUCs were calculated for the entire study population to compare the predictive performance of TBS and BMD at the lumbar spine and femoral neck (Fig. 4). AUCs for TBS, lumbar spine BMD, and femoral neck BMD were 0.700, 0.737, and 0.689, respectively. There were no significant differences in the AUCs for TBS, lumbar spine BMD, and femoral neck BMD.

Table 2 shows the results of univariate analysis of risk factors for vertebral fractures. Age (> 80 years), slimness, a low TBS, and osteoporosis (at the lumbar spine or femoral neck) were significantly associated with vertebral fractures. Multiple logistic regression analysis identified BMD at the lumbar spine (*p* < 0.001, Exp(B) = 120), TBS (*p* < 0.001, Exp(B) = 1241), and female sex (*p* = 0.002, Exp(B) = 3.1) as significant risk factors for vertebral fracture (Table 3).

**Table 2:** Univariate analyses of factors associated with occurrence of vertebral fractures in the entire study population (n = 279)

Variable	OR (95% CI)	***p***-value
Age (years)		
60–69	1.0 (Reference)	
70–79	1.27 (0.59–2.70)	0.57
80–90	3.36 (1.66–6.77)	0.001
Female sex	1.19 (0.70–2.02)	0.59
BMI (kg/m^2^)		
<18.5	1.0 (Reference)	
18.5–25	0.67 (0.32–1.41)	0.67
>25	0.34 (0.14–0.80)	0.02
Type 2 DM	0.86 (0.42–1.80)	0.85
Rheumatoid arthritis	1.28 (0.62–2.64)	0.57
Steroid use	1.56 (0.63–3.88)	0.46
TBS (L2–4)		
High (≥ 1.31)	1.0 (Reference)	
Intermediate (1.23–1.31)	2.00 (1.85–6.50)	0.02
Low (≤ 1.23)	10.56 (4.81–17.10)	< 0.001
BMD (lumbar spine)		
Normal	1.0 (Reference)	
Osteopenia	3.47 (1.85–6.50)	< 0.001
Osteoporosis	8.65 (4.30–17.10)	< 0.001
BMD (femoral neck)		
Normal	1.0 (Reference)	
Osteopenia	1.93 (0.68–5.39)	0.22
Osteoporosis	4.85 (1.83–12.77)	< 0.001

BMD, bone mineral density; BMI, body mass index; CI, confidence interval; DM, diabetes mellitus; OR, odds ratio; TBS, trabecular bone score

**Table 3:** Multiple logistic regression analysis: factors associated with vertebral fractures in the entire study population (n = 279)

Variable	Exp (B)	***p***-value
Female sex	3.1 (1.5–6.4)	0.002
Type 2 DM	-	0.81
Rheumatoid arthritis	-	0.15
Steroid use	-	0.13
TBS (L2–4)	1241 (46–33,132)	< 0.001
BMD (lumbar spine)	120 (16–659)	< 0.001
BMD (femoral neck)	-	0.66

BMD, bone mineral density; DM, diabetes mellitus; TBS, trabecular bone score

**Fig. 5:**
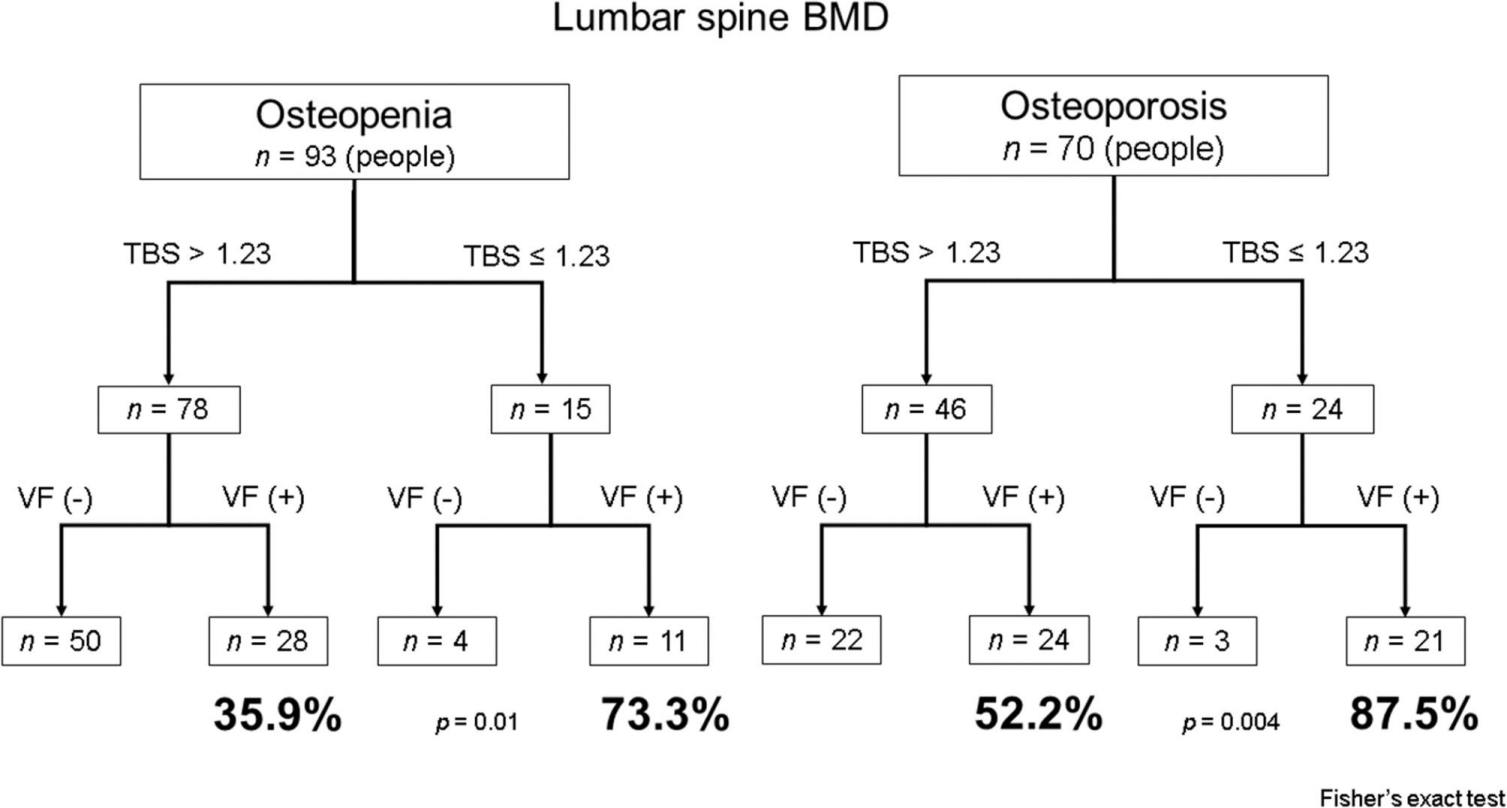
Relationship between TBS and vertebral fractures in lumbar spine BMD in the osteopenia and osteoporosis groups. BMD, bone mineral density; TBS, trabecular bone score; VF, vertebral fractures

When all 279 patients were classified according to lumbar spine BMD, 70 were diagnosed as having osteoporosis and 93 as having osteopenia (Fig. 5). In the osteoporosis group, there were 24 patients with a low TBS (≤ 1.23), 87.5% of whom had vertebral fractures. Of the 46 patients with a non-low TBS (> 1.23) in the osteoporosis group, 52.2% had vertebral fractures. The proportion of patients with vertebral fractures was higher among those with a low TBS than among those with a non-low TBS in both the osteoporosis group (*p* = 0.004) and the osteopenia group (*p* = 0.01).

## Discussion

This study investigated the value of TBS in patients with vertebral fractures. TBS was a significant indicator of vertebral fractures in multiple logistic regression analysis. In the osteoporosis and osteopenia groups, the proportion of patients with vertebral fractures was higher in those with a low TBS than in those with a non-low TBS.

Previous cohort studies have also demonstrated that TBS is a predictor of fracture risk (12,21,28, 33, 34,35,36). The JPOS cohort study showed that the combination of TBS and BMD significantly improved the accuracy of risk prediction compared with BMD alone in Japanese women [35]. A cross-sectional sub-study of NoFRACT found that patients with prevalent vertebral fractures were older and had a lower BMD and TBS than those without vertebral fractures [36]. However, other studies have found less of an association between TBS and fractures (21,27,^37^). For example, the Manitoba study reported that TBS was not associated with incident clinical vertebral fractures in postmenopausal women [21] and a prospective cohort study found no relationship between TBS and incident clinical or radiographic vertebral fractures in older men [27]. There could be an explanation for the inconsistent findings of these studies. First, TBS may vary according to ethnicity. A meta-analysis of 14 cohort studies showed that the mean TBS for postmenopausal women ranged from 1.23 to 1.31 according to ethnic group [32]. In our study, the mean TBS was 1.32, which is almost the same as that in the JPOS cohort study, which was performed in the Japanese population [35]. Therefore, it seems better to limit comparisons between studies to those performed in the same ethnic groups.

In our study, TBS was a significant explanatory factor for vertebral fractures in multiple logistic regression analysis. This finding supports other reports suggesting that TBS is an independent predictor of fracture risk [12, 13, 21, 22, 38]. In addition, the proportion of people with vertebral fractures was higher in patients with a low TBS than in those with a non-low TBS in both the osteoporosis and osteopenia groups, which indicates that a lower TBS is associated with a higher fracture risk [23–25, 39]. It has been reported that a combination of TBS and BMD improves prediction of fractures [11, 25, 35, 40], which is consistent with the present findings.

The relationship between lumbar spine BMD and TBS is shown in Fig. 5. In the osteoporosis group, there was a difference in the incidence of vertebral body fractures due to the difference in TBS, and many people had vertebral fractures. In the osteopenia group, there were also differences in the incidence of vertebral fractures due to the difference in TBS. Therefore, by using TBS, it was possible to improve the ability to identify vertebral fractures. Guidelines published by the American Association of Clinical Endocrinologists and the American College of Endocrinology strongly recommend pharmacologic therapy for patients with osteoporosis or low-trauma spinal fractures (regardless of BMD) [27]. We believe that use of TBS in patients with osteopenia might improve our ability to identify those with vertebral fractures who require pharmacologic therapy.

The primary strength of this study is that patients on medical treatment for osteoporosis were excluded. It is known that TBS and BMD measurements are affected by the drugs used to treat osteoporosis [41–44], and we believe that BMD and TBS could be evaluated more accurately by excluding these patients.

TBS has already been applied in the clinical setting. FRAX was developed by the WHO for predicting and calculating the risk of fractures within the next 10 years. Since 2015, TBS has been combined with FRAX and BMD to calculate the adjusted FRAX probability of fracture in postmenopausal women and older men. The TBS-adjusted probability was more accurate in predicting fractures [28]. The American Association of Clinical Endocrinologists and American College of Endocrinology guidelines strongly recommend pharmacologic therapy for patients with osteopenia if the TBS-adjusted FRAX 10-year probability for major osteoporotic fracture is ≥ 20% or the 10-year probability of hip fracture is ≥ 3% in the US or above the country-specific threshold in other countries or regions [45].

In 2019, the ISCD reported on the usefulness of TBS monitors in determining the therapeutic effect of osteoporosis drugs [46]. The 2019 ISCD position statement considers that the role of TBS in monitoring the efficacy of antiresorptive therapy is unclear and that TBS is potentially useful for monitoring patients on anabolic therapy. This position statement also suggests that a significant decrease in TBS might represent a worsening of trabecular texture, potentially warranting further clinical assessment and a change in treatment strategy. In our study, the proportion of patients with vertebral fractures was lower in those with a non-low TBS. An increase in TBS, which indicates better bone quality, suggests prevention of vertebral fractures. It is known that vertebral fractures and degenerative spondylolysis result in a false increase in lumbar BMD readings on DXA. The efficacy of drug treatment for osteoporosis should be evaluated with a good understanding of the characteristics of TBS and BMD.

There are interesting reports of a new technology that uses DXA images. The bone strain index (BSI), based on finite element analysis, is an innovative index of bone strength that provides information about skeletal resistance to loads not considered by existing indices (BMD, TBS, hip structural analysis [HSA]) [47]. BSI appears to be a useful index for predicting fracture and re-fracture and could be used for more refined risk assessment in patients with osteoporosis [48]. There is no doubt that information on BMD, TBS, and HSA is important; however, BSI based on finite element analysis might also be a useful tool for decision-making regarding treatment of osteoporosis.

This study has several limitations. First, it had a cross-sectional rather than a longitudinal design. Second, the number of patients was relatively small, which might explain why we could not demonstrate a statistically significant increase in fracture risk in patients with rheumatoid arthritis, type 2 diabetes mellitus, and a history of steroid use, which have been reported to be risk factors for fragility fractures [14]. Third, our study population was older than that in some other studies, which could explain the higher proportion of patients with vertebral fractures.

## Conclusion

We investigated the value of TBS in the Japanese population and found it to be a significant indicator of vertebral fracture. The proportion of patients with vertebral fractures was higher in those with a low TBS than in those with a non-low TBS, regardless of whether they had osteoporosis or osteopenia. Use of TBS might improve the ability to identify patients with vertebral fractures in need of pharmacologic therapy, particularly those with osteopenia.

## Electronic supplementary material

Below is the link to the electronic supplementary material.

Supplementary Material 1

## Data Availability

The datasets used and/or analyzed during the current study are available from the corresponding author on reasonable request.

## Abbreviations

AUC: area under the receiver-operating characteristic curve
BMD: bone mineral density
BMI: body mass index
BSI: bone strain index
DXA: dual-energy X-ray absorptiometry
FRAX: Fracture Risk Assessment Tool
HAS: hip structural analysis
ISCD: International Society for Clinical Densitometry
SD: standard deviation
SQ: semiquantitative
TBS: trabecular bone score
VF: vertebral fractures
WHO: World Health Organization
